# Mechanical Performance and Cytotoxicity of an Alginate/Polyacrylamide Bipolymer Network Developed for Medical Applications

**DOI:** 10.3390/ma16051789

**Published:** 2023-02-22

**Authors:** Abdullah Mouchati, Najet Yagoubi

**Affiliations:** Matériaux et Santé, UFR de Pharmacie, Université Paris Saclay, 91400 Orsay, France

**Keywords:** polymer, mechanical properties, medical device, biocompatibility test

## Abstract

Several hydrogels could be used as scaffolds for tissue engineering and a model of extracellular matrices for biological studies. However, the scope of alginate in medical applications is often severely limited by its mechanical behavior. In the present study, the modification of the mechanical properties of the alginate scaffold is obtained by its combination with polyacrylamide in order to obtain a multifunctional biomaterial. The advantage of this double polymer network is due to an improvement in the mechanical strength with regard to the alginate alone, and in particular, its Young’s modulus values. The morphological study of this network was carried out by scanning electron microscopy (SEM). The swelling properties were also studied over several time intervals. In addition to mechanical property requirements, these polymers must meet several biosafety parameters as part of an overall risk management strategy. Our preliminary study illustrates that the mechanical property of this synthetic scaffold depends on the ratio of the two polymers (alginate, polyacrylamide) which allows us to choose the appropriate ratio to mimic replaceable body tissue and be used in various biological and medical uses, including 3D cell culture, tissue engineering, and protection against local shocks.

## 1. Introduction

Biomaterials play an increasing role in modern health care systems. Ideal materials for the engineering of biomimetic tissue should combine several tissue properties, such as mechanical behavior and water content, and could be synthesized to exhibit required biocompatibility [1,2,3,4,5].

In the orthopedic field, the regeneration of cartilage is a real challenge. A significant obstacle is the poor regenerative ability of native cartilage tissue [6,7]. That is why tissue engineering offers a promising opportunity to synthesize artificial cartilage with appropriate mechanical and safety properties [6,8,9].

A three-dimensional porous scaffold is important for cartilage tissue engineering in order to prevent seeded cells from spreading away from the defect site [10,11,12]. Scaffolds also provide cells with the necessary environment for their differentiation and determine the final shape of the engineered cartilage. It should have several additional properties, such as being biocompatible, highly porous, sufficiently robust, and flexible [13].

Hydrogels are considered as an ideal material for the tissue engineering, as they resemble several tissue properties better than other biomaterials. Hydrogels generally provide a suitable environment for cell growth, and they have mechanical and structural properties similar to many tissues. For example, they have a similar high water content and a good biocompatibility; furthermore, they can be manufactured to serve as a synthetic extracellular matrix (ECM) to organize cells into a three-dimensional architecture [5]. Moreover, they can be delivered in a minimally invasive manner.

However, many hydrogels have mechanical limitations, which prevent their usability in medical applications, such as ligament and tendon prostheses or robotic actuators [14,15]. Efforts to overcome these mechanical limitations included the design of hybrid hydrogels. This approach follows the strategy of increasing the hardness of the hydrogel by adding biopolymers or crosslinkers that dissipate energy when strained [16].

Previous studies focused on the combination of the thermosensitive polymers, especially poly(N-isopropyl acrylamide) (PNIPAM) and organometallic complexes, such as triphenyltin chloride [17] and bis(cyclopentadienyl)titanium [18].

In this preliminary study, the combination of a double polymer network between alginate and polyacrylamide was described and revealed suitable mechanical properties [19]. One advantage of this biomaterial is its incorporation of two polymers, alginate and polyacrylamide, the performance and safety properties of which are well-characterized in previous literature.

Alginate is a natural hydrogel used in a broad range of medical applications, especially as the supporting matrix or delivery system for tissue repair and regeneration. Due to its biocompatibility and biodegradability, alginate was widely used in various biomedical applications, including tissue engineering, drug delivery, and in some formulations preventing gastric reflux [15,20,21]. After the approval of the U.S. Food and Drug Administration (FDA), alginate became one of the famous biomaterials in regeneration drugs, nutrition supplements [22], semi-permeable partitions, etc. [23,24,25,26,27,28,29].

Polyacrylamide, a synthetic polymer, was used in supporting electrophoresis in 1959 [22,30]. In Europe and China, polyacrylamide gels were widely used for more than 10 years as implants for reconstructive and cosmetic surgery [16,25,31]. Approximately 30,000 patients were injected with polyacrylamide hydrogel for soft tissue augmentation [22]. During this period, studies indicated that polyacrylamide hydrogel is well tolerated and does not cause any biological reactions. The safety of the polyacrylamide component is well characterized. Kebuladze et al.’s reports show that polyacrylamide hydrogel is well-tolerated in the subcutaneous compartment and glandular breast tissue, and further, that polyacrylamide hydrogel stays in place at the injection site without being degraded or displaced, which is not the case of other products used for augmentation, such as silicone and collagen gel [16,31].

As synthesized alginate/polyacrylamide scaffolds exhibited required mechanical properties, which are close to natural human cartilage tissues and good water uptake, our present study prepared the alginate/polyacrylamide scaffolds in different ratios in order to show the usability of this scaffold in several medical applications without any cytotoxicity effect, especially the toxicities associated with being extractable and leachable that can leach out from this double polymer and potentially cause biological interactions in the body.

## 2. Materials and Methods

### 2.1. Materials

Polyacrylamide, sodium alginate, N, N′-methylene bisacrylamide, tetramethylethylenediamine (TEMED), ammonium persulfate (KPS), and calcium chloride were purchased from Sigma-Aldrich (St. Louis, MO, USA).

### 2.2. Synthesis of Double Polymer Network Alginate/Polyacrylamide

**Figure 1:**
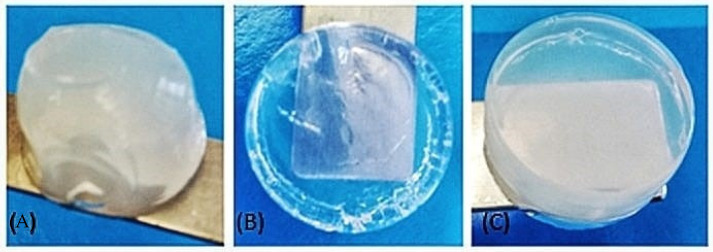
Scaffold images of alginate alone (**A**), polyacrylamide (**B**), and double polymer network alginate/polyacrylamide (**C**).

Double polymer networks at different ratios of ALG/PAAm (4/4, 4/10, 8/20, 10/33, and 15/50%) were fabricated as previously described with polymerization modifications [6,10,13]. The different ratios of ALG/PAAm (4/4, 4/10, 8/20, 10/33, and 15/50%) were homogeneously dissolved in 30 mL of ultra-distilled water. These solutions were rapidly mixed with 0.4% of N, N′-methylene bisacrylamide, 0.04 of tetramethylethylenediamine, and 0.04% of ammonium persulfate, and they were inversed in glass vials. These percentages were used in the case of ALG/PAAM 4/4% scaffold. After degassing the solution with nitrogen gas, the polymerization was initiated by putting the glass vials in an incubator at 60 °C for 2 h. Then, it was ionically crosslinked by calcium cation in 10 wt.% of calcium chloride solution for 24 h (Figure 1, Table 1).

Finally, the scaffold was washed three times in 8 mL of ultra-distilled water for 24 h.

Scaffolds of polyacrylamide alone and alginate alone were also prepared as controls, using the same weight percentage of monomers as in the hybrid scaffolds.

### 2.3. Mechanical Properties—Compression Test

The elastic modulus measurement of double polymer network alginate/polyacrylamide at different percentages (4/4, 4/10, 8/20, 10/33, and 15/50%) was performed by a texture analyzer (TAX-T2; Texture Technologies, Hamilton, MA, USA) at room temperature with a strain rate of 0.1 mm/sec. Young’s Modulus was calculated from the linear region of the stress/strain curve slope. The mechanical test methodology used in this article is close to EN ISO 844:2021 [32]. For testing the mechanical properties of the double polymer network, cylindrical scaffolds 2 cm in diameter and 8 mm in thickness were tested as above. The strain was terminated at 50% of deformation.

The scaffolds of alginate alone and polyacrylamide alone are used in the same previous ratios to serve as a control in the mechanical test.

### 2.4. Preliminary Study

#### 2.4.1. Microstructure Evaluation

A thin film (approximately to 1 mm) of the double polymer network ALG/PAAm scaffold (4/4%) was lyophilized (BETA 2-8, CHRIST, Dusseldorf, Germany) in order to evaluate the microstructure of double polymer network scaffolds with scanning electron microscopy SEM (FlexSEM 1000, HITACHI, Tokyo, Japan) at a beam voltage of 5 kV.

#### 2.4.2. Swelling Test

The mass swelling ratio of alginate alone, polyacrylamide alone and ALG/PAAm (4/4%) scaffolds were measured following the incubation in ultra-distilled water at room temperature. Scaffolds were placed into a 6-well plates containing 8 mL of ultra-distilled water maintained at room temperature. The swelling ratio was calculated by measuring the mass of the scaffolds at 0, 2, 6, and 15, 21 days as follows:

Swelling percentage (%) = (Ms/Mi)100 (1)

where Ms is the mass of the swollen scaffold in ultra-distilled water and Mi is the mass of the initial scaffold before swelling. The reswelling ratios of alginate alone, polyacrylamide alone, and ALG/PAAm scaffolds (4/4%) were measured using the same formula of swelling percentage (1) as a function of time initially equilibrated at ultra-distilled water.

#### 2.4.3. Extractable and Leachable Test

The toxicity of these biomaterials often comes from leachable and extractable components. Extractable and leachable are chemical compounds. The extractable is generated from the material under aggressive conditions using specified experimental conditions (i.e., solvent, time, temperature, humidity, and pressure), while leachable are expected to be present in a medical device under clinical use conditions [33,34,35].

ISO 10993 provides guidance on the requirement biological safety tests that should be performed to get a CE mark. The biological evaluation of a medical device is a part of the risk management process, which should begin with collecting a chemical characterization of the material identified in the medical device. In this study, the monomers, crosslinker, catalyzer, and initiator were the components of the double polymer network. These components were considered as extractable, which were characterized by size exclusion chromatography (SEC) with a multi solvent column (PL Multisolvent 20, 4.6 mm × 150 mm, Agilent technologies, St. Clara, CA, USA) and pre-column (PL Multisolvent, 20 4.6 mm × 50 mm, Agilent Technologies). These components were injected into the system and then eluted using an isocratic flow (NaH_2_PO_4_ 25 mM, Na_2_HPO_4_ 25 mM, NaCl 0.1 M in ultra-distilled water, buffered at pH 7.84) at a flow rate of 0.3 mL·min^−1^ and a set temperature of 30 °C. The scaffold of alginate/polyacrylamide (4/4%) was incubated in ultra-distilled water for 96 h at room temperature. The extract solution was lyophilized and then the mobile phase (NaH_2_PO_4_ 25 mM, Na_2_HPO_4_ 25 mM, NaCl 0.1 M in ultra-distilled water, buffered at pH 7.84) was added to the lyophilized extract solution in order to dissolve the residuals monomers [36].

#### 2.4.4. In Vitro Cytotoxicity Assessment

This preliminary test aims to screen the safety of the double polymer network using mammalian cell cultures. It is one of the oldest assays explicitly designed to screen plastics for toxicity [26,37].

Cytotoxicity of the scaffold of alginate/polyacrylamide (4/4%) was assessed with cell culture methods according to ISO 10993-5 guidelines with a direct contact method and extracted method using MTT assay.

#### 2.4.5. Cell Culture

Mouse fibroblastic connective tissue cells—L929 (C3H/An, Sigma®, Kawasaki City, Japan) were cultured in Dulbecco’s modified eagle’s medium (DMEM, Sigma^®^), supplemented with 10% fetal bovine serum (FBS, Sigma^®^) and 1% antibiotics (100 U/mL penicillin (Sigma^®^) and100 μg/mL streptomycin (Sigma^®^). Eight independent samples were used for each test. The cells were seeded in wells (96-well plates), then scaffold or extracted solution was deposited onto the cells as detailed below [38].

#### 2.4.6. MTT Assay

The MTT assay is a colorimetric assay, as it can be rapidly performed on a microtiter plate assay and read on a spectrophotometer plate reader at the absorbance of 570 nm; that is why it is used to determine the cell viability and the cytotoxicity effect of the materials.

Currently, in the MTT assay, the tetrazolium MTT (3-[4,5-dimethylthiazol-2-yl]-2,5-diphenyltetrazolium bromide) is reduced in a mitochondrial-dependent reaction to an insoluble purple formazan by cleavage of the tetrazolium ring by succinate dehydrogenase within the cell since it cannot pass through the cell membrane. The formazan is solubilized and liberated by adding spectrophotometric-grade dimethylsulfoxide (DMSO) or other suitable solvents. The formazan will be ready to be quantified colorimetrically. Cytotoxic concentration is generally determined as the concentration that kills 50% of the cells, generally known as the IC50 [38].

The direct contact method, which is the direct contact of the biomaterial onto a culture layer, is more sensitive than the rabbit intramuscular implantation test, but care must be taken to avoid physical damage to the cells by pressure or movement of the sample.

The extracts method dilutes the material in cell culture media and provides a quantitative comparison with reference extracts.

After 24 h of fibroblasts seeding into 96-well plates at a 40,000 cells/well density, the direct method was applied, where double polymer network (alginate\polyacrylamide) scaffolds (2.5 mm × 3.5 mm) were gently deposited on cell monolayers and were kept in contact for 24, 48, 72, and 96 h at 37 °C–5% CO_2_. The extracted method was conducted by incubating the scaffold in the culture medium for 72 h at 37 °C–5% CO_2_ and then this extract solution was incubated with the cultured cell 96-well plate for 48 h at 37 °C–5% CO_2_. After changing the culture medium, 20 μL of MTT (Sigma^®^) solution (5 mg/mL in PBS) was then added to each well and incubated for a further 2 h at 37 °C. Then, the MTT solution with the culture medium was eliminated and 200 μL of dimethyl sulfoxide (DMSO) was added to each well. After 20 min of agitation, the absorbance values were recorded at 570 nm using a microplate reader (ELx800 Bio Tek, Wusseki, VT, USA).

## 3. Results and Discussion

### 3.1. Characterization of Biomaterial Scaffolds

**Figure 2:**
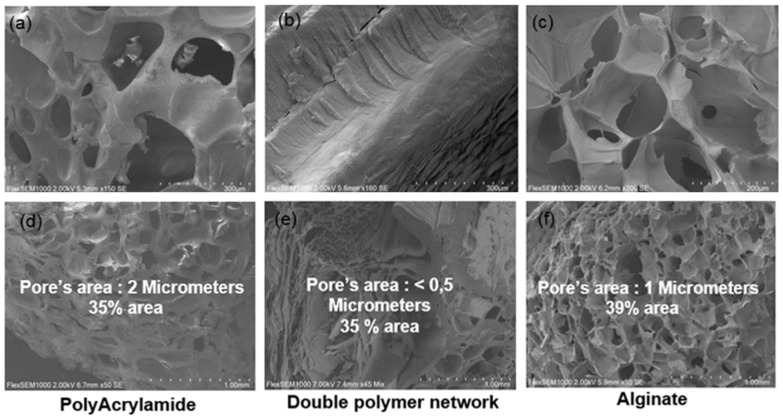
SEM images of polyacrylamide alone, alginate alone, and alginate/polyacrylamide scaffold (4/4%) at scale bars 300µm (**a**–**c**) and scale bars 1 mm (**d**–**f**).

Representative images of synthesized scaffolds of alginate alone, polyacrylamide alone, and the double polymer network ALG/PAAM (4/4%) are shown in Figure 1. Scanning electron microscope (SEM) images of alginate alone, polyacrylamide alone, and alginate/polyacrylamide (4/4%) scaffolds were used to display the three-dimensional, interconnected porous nature of the scaffold (Figure 2). The pore size of alginate alone, polyacrylamide alone, and double network ALG/PAAM scaffolds are 1 micrometer, 2 μm, and 0.5 μm, respectively, based on the SEM images. As shown in the microstructure evaluation by SEM, the double polymer network has the smaller size of the pores, for this reason, a swelling ratio was measured in order to verify the water absorption ability of the double polymer network, and the swelling ratio is considered as the indicator of the capacity of the scaffold to uptake water.

**Figure 3:**
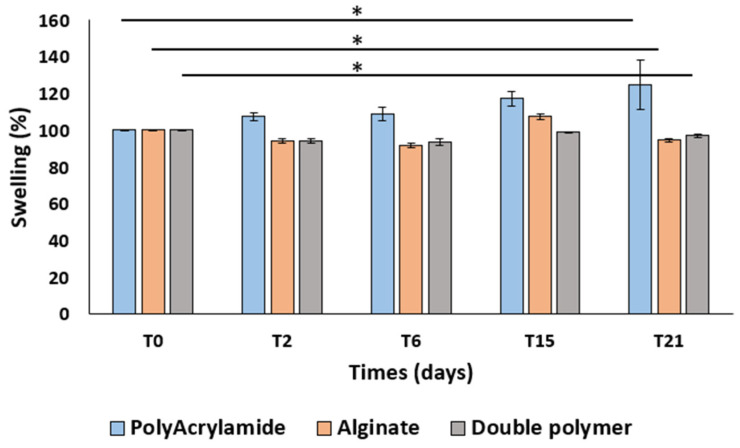
Swelling test: graph shows the swelling percentage (%) of the alginate scaffold, polyacrylamide scaffold, and double polymer network (alginate\polyacrylamide 4\4%) scaffold (* statistical difference *p* < 0.092 for the polyacrylamide scaffold, *p* < 0.013 for the alginate scaffold, and *p* < 0.005 for the double polymer) (*n* = 3).

Figure 3 shows that the double polymer network did not significantly impact the water uptake capacity compared to scaffolds of alginate alone and polyacrylamide alone.

### 3.2. Mechanical Properties

**Figure 4:**
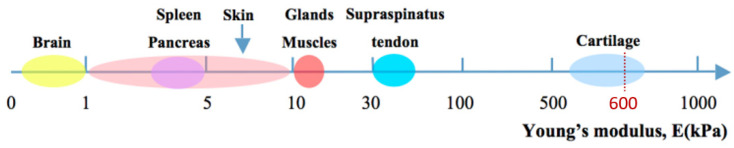
Young’s modulus of natural soft tissues and organs in kPa [5].

The most common mechanical property of hydrogels for tissue engineering is their Young’s modulus, an independent measure of stiffness and elastic properties. According to the function and location of the tissues, Young’s modulus values for native soft tissues and organs range from 0.1 kPa to 1 MPa (Figure 4) [5,39,40,41]. The typical hydrogel scaffolds for artificial cartilage or other medical applications should offer an appropriate stiffness, mimicking the proposed native tissues and allowing them to be cast, support cell growth, and maintain tissue architecture for a while [42]. The hardness of hydrogels could be changed by the polymer concentration, physical or chemical crosslinking, increasing the degree of crosslinking, removing divalent metal ions, and adjusting environmental conditions (e.g., pH value, temperature) [43,44]. For example, Bryant et al. increased polymer concentration from 10% to 20% to fabricate PEG-based hydrogels with Young’s moduli ranging from 60 to 500 kPa [45].

This preliminary study focusses on the impact of the alginate/polyacrylamide ratio on the young modulus and the possibility to use this double polymer network as artificial cartilage. For this reason, several ratios of alginate/polyacrylamide (4/4, 4/10, 8/20, 10/33, and 15/50%) were evaluated by texture analysis.

**Figure 5:**
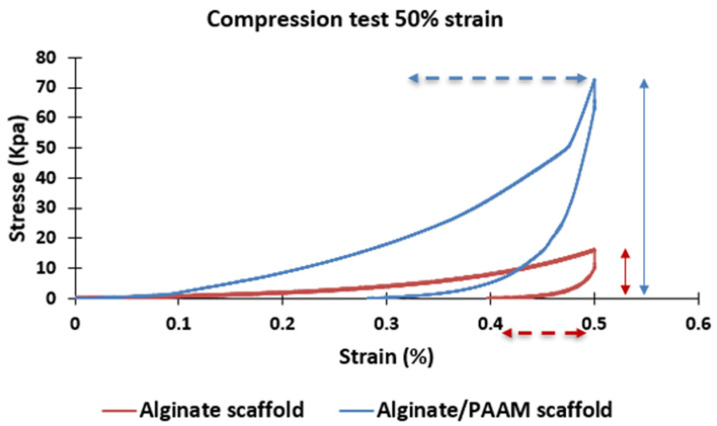
Compression test at 50% of strain for the alginate/polyacrylamide scaffold (blue), and alginate scaffold (yellow).

Figure 5 shows the behavior of alginate scaffold and double polymer network (alginate/polyacrylamide 4/4%) scaffold, and there is a significant increase in flexibility and elasticity of alginate in the presence of polyacrylamide.

**Figure 6:**
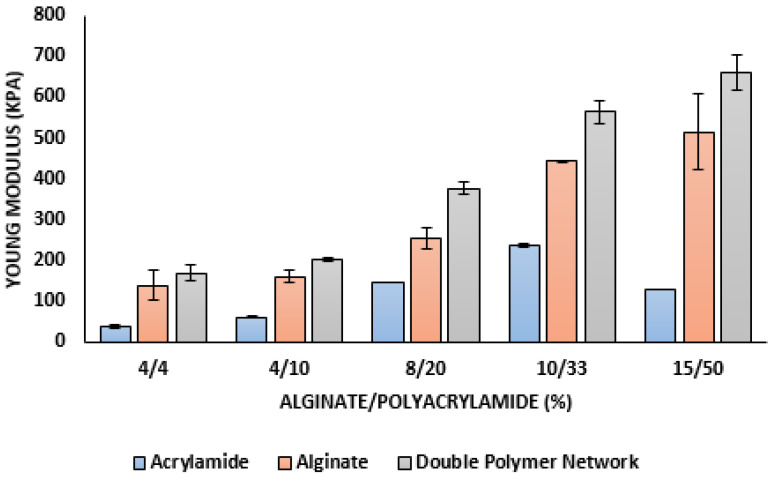
Young Modulus for the alginate scaffold, polyacrylamide scaffold and double polymer network (alginate/polyacrylamide) in several ratios (4/4, 4/10, 8/20, 10/33, and 15/50%) Il manque la barre d’erreur sur le 8/20 et le 15/20 acrylamide.

Figure 6 indicates how the Young modulus value increases with the scaffold of a double polymer network compared with the scaffold of alginate alone and polyacrylamide alone and the progressive increases of Young modulus value with the increasing of the alginate/polyacrylamide ratios. As a result, the 15/50% double polymer network has a Young’s modulus similar to that of cartilage, which is why this scaffold can be used as an artificial cartilage.

As was already seen, the double polymer network’s pore size is smaller than alginate alone and polyacrylamide alone. However, for this type of copolymer, a compromise should be made between the surface state, surface porosity, and mechanical properties, as it shows that the mechanical properties for alginate and polyacrylamide alone are not impressive. On the other hand, we increased the young modulus value by the double polymer network.

### 3.3. Preliminary Study

#### 3.3.1. Extractable and Leachable Test

**Figure 7:**
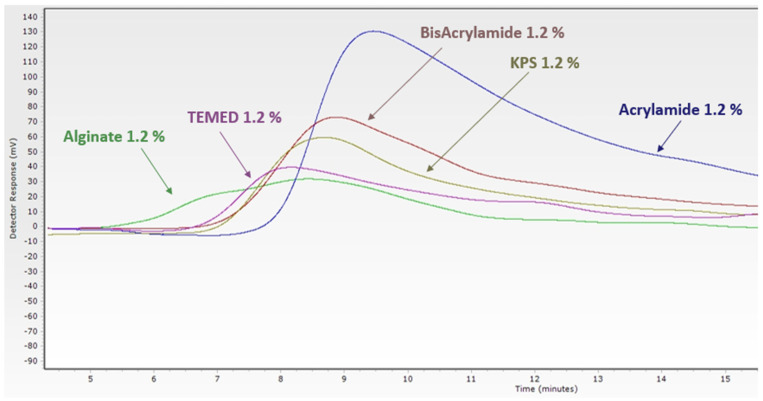
Size exclusion chromatography for the monomers alginate (green) polyacrylamide (purple), crosslinker BisAcrylamide (brouwn), catalyzer TEMED (pink), and the initiateur potasssium peroxodisulfate (kps) (light brown).

For this type of material, extractives and leachable could exist; therefore, the biological safety will depend on the leachable, which must be evaluated before assessing the material biocompatibility. In this study, the residual monomers were considered as leachable, that is why the refractive index (RI) of each monomer is shown in Figure 7 by the size exclusion chromatography.

**Figure 8:**
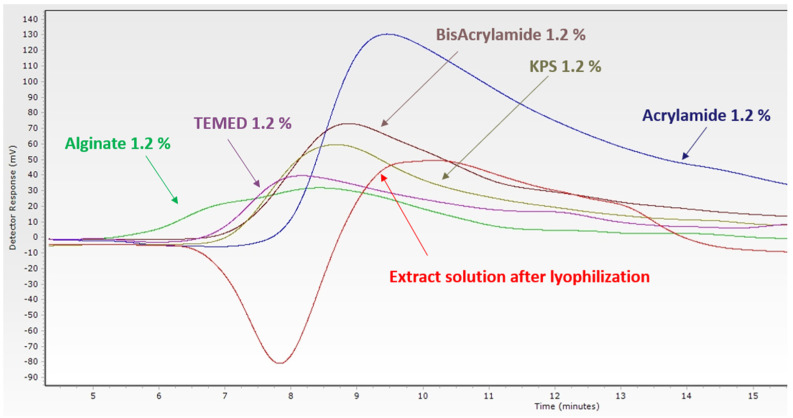
Size exclusion chromatography for the monomers alginate (green) polyacrylamide (purple), crosslinker bisacrylamide (brown), catalyzer TEMED (pink), and the initiateur potassium peroxodisulfate (kps) (light brown) extracted solution of double polymer network (red).

In order to measure the presence of residual monomers, the extracted solution of the scaffold was injected in size exclusion chromatography and a single peak was highlighted at the same point of polyacrylamide’s elution time, which is 9 min (Figure 8); that is why the monomers of polyacrylamide were considered as residual monomers. These residual monomers could have a toxic effect; therefore, a cell reactivity test will be conducted to evaluate the toxicity effect of the residual polyacrylamides.

#### 3.3.2. Cytotoxicity

**Figure 9:**
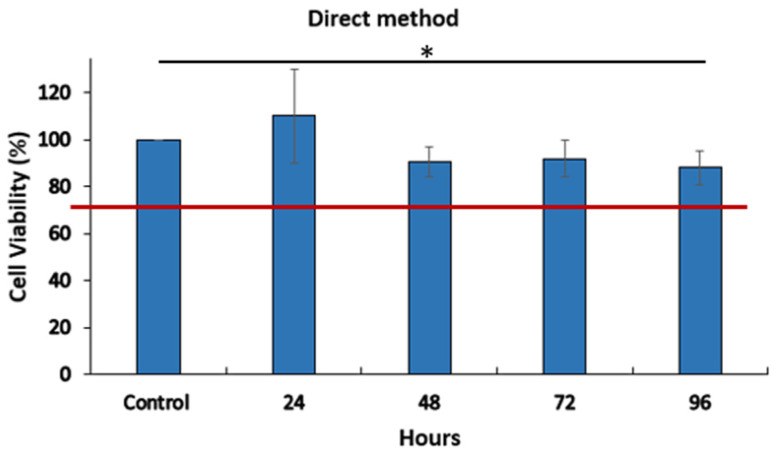
Cytotoxicity test, direct method (* statistical difference *p* < 0.05) (*n* = 3).

**Figure 10:**
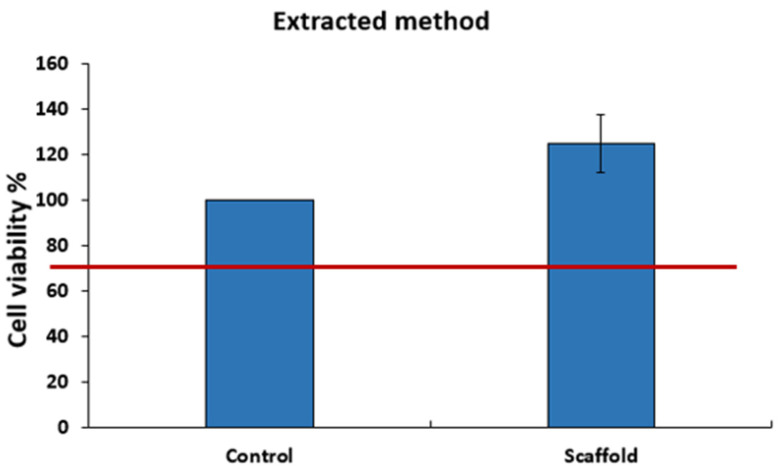
Cytotoxicity test, extracted method.

The mitochondrial activity (MTT), measured after 24, 48, 72, and 96 h of exposure to the double polymer network (4/4%), shows no significant reactivity of L929 fibroblasts (Figure 9). This result was confirmed with the extract’s method when the MTT activity was measured after 48 h of exposure to the extracted solution (Figure 10). All results were above 70% of cell viability, which is the normative specification. These results prove the cell viability in contact with the leachable and direct contact with the scaffold. These observations proposed the absence of the cytotoxicity effect of the double polymer network alginate/polyacrylamide (4/4%) for L-929 cells.

**Figure 11:**
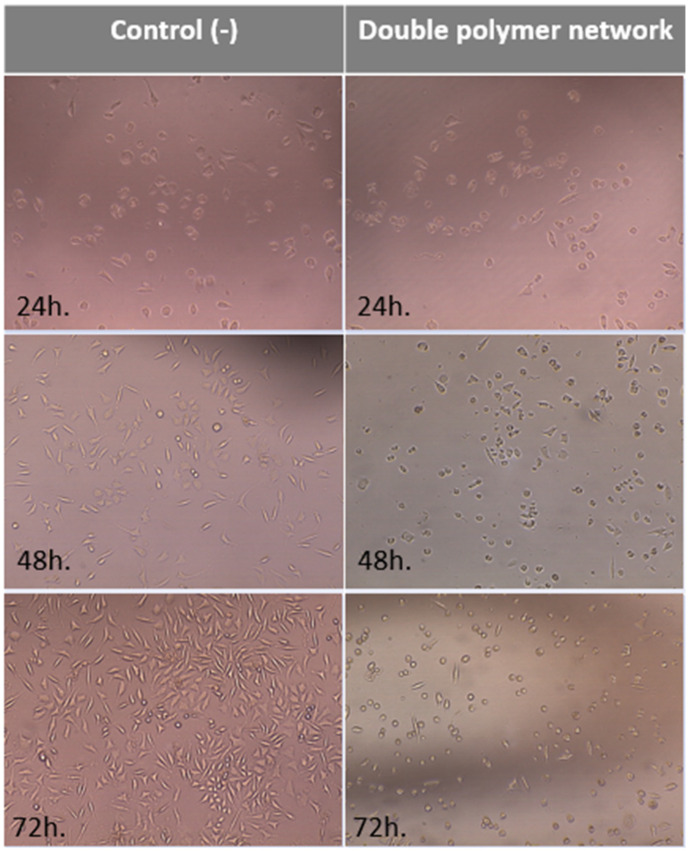
Exemplary photographs of mouse fibroblasts cells (L-929), with negative control (left) and extract solution of the double polymer network (right) at several intervals of time (24, 48, and 72 h.) (phase contrast micrographs, 100×).

In addition to the cell viability, the cell morphology showed no cell lysis, many cells in fission, and the density of culture is comparable to the density of negative control culture (Figure 11).

## 4. Conclusions

The mechanical properties of the synthesized alginate/polyacrylamide scaffolds can be changed by changing the ratios of alginate/polyacrylamide (4/4, 4/10, 8/20, 10/33, and 15/50%). As seen before, the alginate/polyacrylamide scaffold corresponding to the ratio 15/50%, respectively, has a young modulus close to the native cartilage tissue. This result justifies the usability of this double polymer material. These preliminary results suggest a good candidate for cartilage replacement, and they open perspectives to other medical applications. Furthermore, we are encouraged to believe in this double polymer scaffold, which holds much promise for future studies in various biological and medical applications, including 3D cell culture, and cartilage and bone tissues engineering.

## Figures and Tables

**Table 1:** The prepared samples.

ALG/PAAM	Alginate Percentage (%)	Polyacrilamide Percentage (%)
4/4	4	4
4/10	4	10
8/20	8	20
10/33	10	33
15/50	15	50

## Data Availability

Not applicable.
